# Estimating Grassland Carbon Stocks in Hulunber China, Using Landsat8 Oli Imagery and Regression Kriging †

**DOI:** 10.3390/s19245374

**Published:** 2019-12-05

**Authors:** Lei Ding, Zhenwang Li, Xu Wang, Ruirui Yan, Beibei Shen, Baorui Chen, Xiaoping Xin

**Affiliations:** 1 National Hulunber Grassland Ecosystem Observation and Research Station, Institute of Agricultural Resources and Regional Planning, Chinese Academy of Agricultural Sciences, Beijing 100081, China; dinglei0206@126.com (L.D.); wangxu01@caas.cn (X.W.); yanruirui19790108@163.com (R.Y.); 13121265299@163.com (B.S.); chenbaorui@caas.cn (B.C.); 2 Institute of Soil Science, Chinese Academy of Science, Nanjing 210008, China; zwli@issas.ac.cn

**Keywords:** carbon stocks, grassland, regression kriging, Landsat8 OLI

## Abstract

Accurately estimating grassland carbon stocks is important in assessing grassland productivity and the global carbon balance. This study used the regression kriging (RK) method to estimate grassland carbon stocks in Northeast China based on Landsat8 operational land imager (OLI) images and five remote sensing variables. The normalized difference vegetation index (NDVI), the wide dynamic range vegetation index (WDRVI), the chlorophyll index (CI), Band6 and Band7 were used to build the RK models separately and to explore their capabilities for modeling spatial distributions of grassland carbon stocks. To explore the different model performances for typical grassland and meadow grassland, the models were validated separately using the typical steppe, meadow steppe or all-steppe ground measurements based on leave-one-out crossvalidation (LOOCV). When the results were validated against typical steppe samples, the Band6 model showed the best performance (coefficient of determination (R^2^) = 0.46, mean average error (MAE) = 8.47%, and root mean square error (RMSE) = 10.34 gC/m^2^) via the linear regression (LR) method, while for the RK method, the NDVI model showed the best performance (R^2^ = 0.63, MAE = 7.04 gC/m^2^, and RMSE = 8.51 gC/m^2^), which were much higher than the values of the best LR model. When the results were validated against the meadow steppe samples, the CI model achieved the best estimation accuracy, and the accuracy of the RK method (R^2^ = 0.72, MAE = 8.09 gC/m^2^, and RMSE = 9.89 gC/m^2^) was higher than that of the LR method (R^2^ = 0.70, MAE = 8.99 gC/m^2^, and RMSE = 10.69 gC/m^2^). Upon combining the results of the most accurate models of the typical steppe and meadow steppe, the RK method reaches the highest model accuracy of R^2^ = 0.69, MAE = 7.40 gC/m^2^, and RMSE = 9.01 gC/m^2^*,* while the LR method reaches the highest model accuracy of R^2^ = 0.53, MAE = 9.20 gC/m^2^, and RMSE = 11.10 gC/m^2^. The results showed an improved performance of the RK method compared to the LR method, and the improvement in the accuracy of the model is mainly attributed to the enhancement of the estimation accuracy of the typical steppe. In the study region, the carbon stocks showed an increasing trend from west to east, the total amount of grassland carbon stock was 79.77 × 10^4^ Mg C, and the mean carbon stock density was 47.44 gC/m^2^. The density decreased in the order of temperate meadow steppe, lowland meadow steppe, temperate typical steppe, and sandy steppe. The methodology proposed in this study is particularly beneficial for carbon stock estimates at the regional scale, especially for countries such as China with many grassland types.

## 1. Introduction

Grassland ecosystems, which cover some 40% of the terrestrial surface, make up one of the most important and widely distributed terrestrial ecosystems and play an important role in biodiversity conservation and soil protection as well as in global carbon cycle and climate regulation [1,2,3,4]. They also provide important resources to modern society, especially for developing countries [5]. Grassland is a highly complex biome, whose species composition, density and biomass vary greatly with time and space, making it sensitive to changes of soil conditions, local management, climate and weather conditions [6]. Unfortunately, due to high-intensity use, cultivated land reclaimation, and climate variability, grasslands have been degraded worldwide in the past decades [7,8,9,10]. It is reported that more than 90% of the grassland in Inner Mongolia, which is characterized by a semiarid inland climate, is facing serious degradation, with the quality and area of pasture grass decreasing [11].

In pasture areas, the amount of grassland carbon stocks determines the forage availability and herbivore carrying capacity [12,13,14]. Timely and accurate monitoring of the amount of grassland carbon stocks can provide scientific data that can be used to regulate stocking rates for the sustainable use of grassland resources [15,16]. Traditional methods used to estimate carbon stocks are mainly conducted through field surveys, but even though they can provide a better estimation of vegetation carbon stocks, they are too labor- and time-intensive over large areas [17,18] which limits their use [19,20]. Remote-sensing data, which have high temporal resolution and the capacity for large-scale observation, are widely used for large-area carbon stock estimations and can be the most effective means of scaling up grassland carbon stocks from the sample scale to the regional scale [21].

A number of carbon stock estimation methods have been developed to ues remote-sensing data [19,21,22,23,24,25,26,27,28,29,30,31,32,33,34,35,36,37,38,39,40]. The most popular and commonly used approaches are empirical statistical methods [18,21,22,23,26,31], which link various predictor variables derived from remotely sensed data to carbon stock values measured at the ground. Another widely used type of approach is machine-learning methods, such as artificial neural networks [18,31,35,36], support vector machines [37], and random forests [31,32,38,39,40]. Unlike regression methods, these approaches can easily handle a large number of explanatory variables derived from remotely sensed and ancillary data that are linearly or nonlinearly related to biomass [41]. Geostatistical prediction methods, including ordinary kriging (OK) [42], universal kriging (UK) [42,43], and regression kriging (RK), which model the data structure through spatial autocorrelation and incorporate this information into the response variables of unsampled locations [44], have been widely studied for the interpolation of meteorological data and spatial distributions of soil carbon stocks, and they have also been used to map environmental variables [19,45,46,47,48,49]. But there is still a lack of research on the remote-sensing inversion of vegetation carbon stocks, especially grassland carbon stocks. Recent studies have shown the superiority of RK when compared to the other two methods [45,46]. In the study area, RK performs better than the other machine learning methods (ANN and RF) in terms of predicting grassland leaf area index values across the duration of the growing season [45].

Carbon stocks have been estimated using a variety of sources of remote-sensing data. The spaceborne signal aperture radar (SAR) is widely used for the estimation of forest above-ground biomass (AGB) [50,51,52], because it can work under dark and bad-weather conditions. Lidar is an active remote-sensing technology which determines the distance between the sensor and the target using laser energy [40]. Lidar is able to provide accurate information on the vertical structure of forests through recorded discrete returns or waveforms [53,54,55]. The height of vegetation during the nongrowing season of a meadow steppe in northeast China was estimated using lidar data acquired by unmanned aerial vehicles (UVAs) [56]. Passive optical remote sensing provides perhaps the best tool for biomass estimation at regional to global scales because of its global coverage, multiple spatial resolutions, repeat visits, and cost-effectiveness [41]. Landsat-series satellites, as medium-resolution satellites, are widely used in areas of ecological monitoring. The operational land imager (OLI) carried on Landsat8 includes 9 bands with a spatial resolution of 30 m. The OLI includes all the bands of the Enhanced Thematic Mapper+ (ETM+) sensor and adjusts OLI Band5 (0.845–0.885 microns) to exclude the water vapor absorption characteristics at 0.825 microns. In addition, there are two new bands: deep blue Band1 (0.433–0.453 microns), which mainly applies to coastal-zone observations, and short-wave infrared (SWIR) Band9 (1.360–1.390 microns), which includes strong water vapor absorption characteristics and can be used for cloud detection. Near-infrared Band5 and SWIR Band9 are close to the bands corresponding to MODIS [57,58,59]. Vegetation indexes (VIs) are derived based on the combination of multispectral bands of optical satellites are becoming more frequently used in biomass estimation because they can enhance green vegetation signals and minimize the impacts from the soil background, Sun-canopy-sensor geometry, and atmosphere [41]. The normalized difference vegetation index (NDVI), enhanced vegetation index (EVI), simple ratio (SR), wide dynamic range vegetation index (WDRVI), and chlorophyll-based indexes (e.g., chlorophyll index (CI)) are the most commonly used VIs [60].

To better estimate grassland carbon stocks, this study used the RK method to build models incorporating spectral bands and VIs from Landsat8 OLI images. First, we analyzed the correlation between seven bands and five commonly used VIs (NDVI, WDRVI, CI, EVI and SR) and carbon stocks, and we selected the highly correlated parameters as the prediction variables. Then, these variables were used for RK modeling and validation based on ground measurements. A linear regression (LR) model was also built for each variable to compare the results with those of the RK method. Finally, the carbon stock characteristics of the study area were analyzed using the model simulation results with the highest accuracies. The methodology proposed in this study is particularly beneficial for carbon stock estimates at the regional scale, especially for countries such as China with many grassland types. The first draft of this article used the RK method to estimate grassland AGB based on the Landsat8 OLI images, band4, NDVI, and EVI, which were used to build RK models separately and to explore their capability for modeling spatial distribution of grassland AGB [61]. In this study, we have made improvements in the following aspects: (1) In terms of the methods, two databases of typical grasslands and meadow grasslands were added for validation respectively, to explore the model performance. (2) To obtain more accurate models, the WDRVI, the CI, the SR and other spectral bands were added as regression parameters. (3) We converted the aboveground biomass into carbon stocks. (4) We enriched the contents of the Introduction, Results, and Discussion sections.

## 2. Methods and Data

### 2.1. Study Area

**Figure 1:**
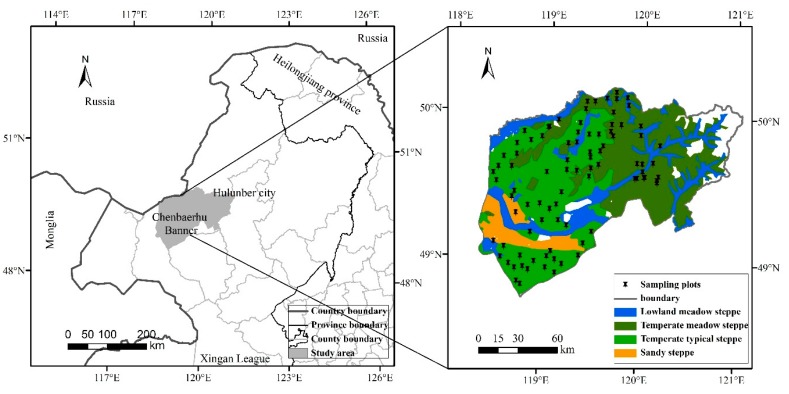
Study area and sampling plots.

Chenbarhu Banner is located in the backland of Hulunber (48°48′–50°12′ N, 118°22′–121°02′ E) in the northeastern part of Inner Mongolia, China, and is part of the Mongolian Plateau in central Asia (Figure 1). This region is characterized by a semiarid inland climate, with an annual mean precipitation of 300–550 mm and an annual mean temperature of about 1 °C [62]. This region is vulnerable and sensitive to climate change, and the average elevation is 677 m, with the elevation and rising from west to east. The length of the growing season is approximately 140 days and lasts from May to September [63]. Grasslands are the largest ecosystem in this region, and four main grassland types are included: lowland meadow steppe, temperate meadow steppe, temperate typical steppe and sandy steppe. Among them, temperate typical steppe and sandy steppe are typical steppes, and lowland meadow steppe and temperate meadow steppe are meadow steppes. The total area of the available steppe is approximately 1.68 × 10^6^ hm^2^, in which the area of meadow steppe is 0.93 × 10^6^ hm^2^ and the area of typical steppe is 0.75 × 10^6^ hm^2^.

### 2.2. Sample Design and Measurement of Field Carbon Stocks

A field survey was performed from 9 July to 16 July in 2015. The 1:1,000,000 digital vegetation map of the People’s Republic of China [64] was used to guide the selection of the location of the survey plots relative to the different vegetation types, including typical steppe, meadow steppe, swamp, crops, and broadleaf deciduous forest. Each survey sample plot was 30 m × 30 m and had a homogeneous canopy, which was well-suited for the 30 m-resolution Landsat data. Three 1 m × 1 m quadrats were randomly selected in each sample plot [39]. The above ground biomass is defined as the dry weight of all aboveground live mass per unit area [65], and provides the basis upon which to estimate the aboveground net primary productivity [66]. Usually, for forest ecosystems, the carbon content or conversion coefficient from biomass to carbon storage is 0.5 [67]. For grasslands, we use a value of 0.45, as mentioned in [23,68]. The grassland AGB was obtained using the harvest method [39]. First, fresh grass was cut from ground with stubble no taller than 0.5 cm, and the fresh grass was brought to the laboratory. The fresh grass was dried at 65 °C for 48 h in the oven, and the weight was measured and recorded. A sample plot’s AGB is the average weight of three quadrats. A handheld differential global positioning system (GPS) receiver capable of providing real-time positioning with 2-m accuracy was used to obtain the coordinates of these sample plots. In total, data from 84 sample plots were obtained, including 55 typical steppe samples and 29 meadow steppe samples.

### 2.3. Satellite Data Collection and Processing

Landsat8 OLI lever-1 standard data products [58] were acquired from the United States Geological Survey (USGS) Earth Explorer website (http://earthexplorer.usgs.gov/). The images were radiometrically and geometrically corrected and were projected as UTM coordinates (WGS84 datum, Zone 51 N). To cover the study area, four scenes of Landsat8 images corresponding to the dates of the field survey were collected in this study. Two of the images (Path 123, rows 25 and 26) were acquired on 5 July, and the other two scenes (Path 124, rows 25 and 26) were acquired on 12 July. All images were of high quality and had minimal (<10%) or no cloud contamination (Table 1). To obtain the reflectance of the top of the canopy (TOC), the images were atmospherically corrected using the Fast Line-of-sight Atmospheric Analysis of Spectral Hypercubes (FLAASH) program embedded in ENVI 4.8 software [69].

Five vegetation indexes were calculated based on the TOC reflectance images and used in this study, which were the NDVI [70], the WDRVI [71], the CI [72,73], the EVI [74] and the SR [75]. The indexes were computed using the following equations:

(1) NDVI=(NIR−R)/(NIR+R)

(2) $$WDRVI=\frac{\alpha NIR-R}{\alpha NIR+R}$$

(3) CI=NIR/G−1

(4) $$EVI=\frac{2.5\left(NIR-R\right)}{NIR+C_{1}R-C_{2}B+L}$$

(5) $$SR=\frac{NIR}{R}$$

where *B*, *G*, *R*, and NIR refer to the reflectances in the blue, green, red and near-infrared bands, respectively. In this study, a value of 0.1 is used for the weighting coefficient α [65], *L* = 1, *C*_1_ = 6, and *C*_2_ = 7.5, because the values calculated according to the WDRVI formula are very small and mostly negative, so a value of 0.9/1.1 is added during modeling.

### 2.4. Regression Kriging

RK is a hybrid geostatistical method that combines the LR method with ordinary kriging of the residuals [28]. It is a powerful spatial prediction technique that can be used to interpolate sampled environmental variables (both continuous and categorical) from large point sets [44]. In the process of RK, two parts of the predictions are combined—one is the predictive trend (obtained by regressing the primary variable on the auxiliary predictor using LR), and the other is the residuals, which are interpolated using OK [19]. OK has been proven to be a very reliable and accurate interpolation method [76]. Finally, predictions at unvisited locations ${\hat{z}}_{RK}\left(s_{0}\right)$ are performed by summing the predicted trend and residuals [43]:

(6) $${\hat{z}}_{RK}\left(s_{0}\right)=\overset{p}{\underset{k=0}{\sum }}{\hat{\beta }}_{k}q_{k}\left(s_{0}\right)+\overset{n}{\underset{i=1}{\sum }}\lambda_{i}e\left(s_{i}\right)$$

Here, ${\hat{\beta }}_{k}$ corresponds to the estimated trend model coefficients; *q_k_*(*s*_0_) represents the predictive variables at location *s*_0_; *p* is the number of auxiliary predictors or variables, with common auxiliary environmental predictors including land surface parameters, remote-sensing images, and geological, soil, and land-use maps [77]; *e*(*s_i_*) is the residual of the regression model at site *s_i_*; *λ_i_* is the kriging weight determined by the spatial autocorrelation structure of the residual; and *n* is the number of known points used to estimate the unknown points. The predicted and residual values were calculated, as the structure of the regression model was established for both Landsat8 dates to ensure that these values met the assumptions of normality and homoscedasticity. The structure of the empirical variograms was also determined according to these datas through tests. Three theoretical variograms (exponential, Gaussian, and spherical) [78] were also assayed. The analysis was accomplished using the “gstat” package [79] within the statistical software package R 3.3.3.

### 2.5. Model Assessment

The carbon stock estimation models were built using data from all of the field samples, with samples of typical steppe and meadow steppe validated separately. Cross-validation can be used to compare the performances of different predictive modeling procedures [80], and it is especially suitable for small samples. As a special type of cross-validation, LOOCV provides an almost unbiased estimate of the generation error and can be considered to provide reliable criteria for parameter selection [81,82,83]. To obtain relatively accurate verification results, LOOCV was used in the model assessment. The coefficient of determination (R^2^), mean average error (MAE), and root mean square error (RMSE) were used to determine which models had more precision in the estimation of grassland carbon stocks:

(7) $$R^{2}=1-\frac{\sum_{i=1}^{n}{\left(y_{i}-\hat{y_{i}}\right)}^{2}}{\sum_{i=1}^{n}{\left(y_{i}-\bar{y_{i}}\right)}^{2}}$$

(8) $$MAE=\frac{1}{n}\overset{n}{\underset{i=1}{\sum }}(\left|y_{i}-{\hat{y}}_{i}\right|)*100\%$$

(9) $$RMSE=\sqrt{\frac{1}{n}\overset{n}{\underset{i=1}{\sum }}{\left(y_{i}-{\hat{y}}_{i}\right)}^{2}}$$

Here $\hat{y_{i}}$ is the predicted carbon stock value, *y_i_* is the measured carbon stock value, $\bar{y_{i}}$ is the measured mean values of carbon stock, and *n* is the number of measured values in the validation data.

## 3. Results

### 3.1. Field Carbon Stock Measurements

The detailed summary statistics of the carbon stocks are shown in Table 2. According to the Shapiro-Wilk normality test, the three databases all conform to a normal distribution.

### 3.2. Correlation Analysis

The linear correlation analysis (Pearson) is shown in Table 3. All vegetation indexes were significantly correlated with the carbon stocks (*p* < 0.01). For all-steppe and meadow steppe samples, the CI was more noticeable based on its highest positive coefficient value, followed by the WDRVI. For the typical steppe samples, the NDVI showed the strongest correlation with carbon stocks, followed by the WDRVI. In terms of spectral bands, Band4 (red), Band6 (SWIR1) and Band7 (SWIR2) had the highest correlations, respectively, for the all-steppe, typical steppe and meadow steppe samples. Band5 (near-infrared) had a very low correlation with the carbon stocks of the typical steppe. After the above analysis, we finally chose the NDVI, the WDRVI, the CI, Band6 and Band7 as predictive variables. Compared with other variables, they were highly correlated with the measured carbon stocks.

**Figure 2:**
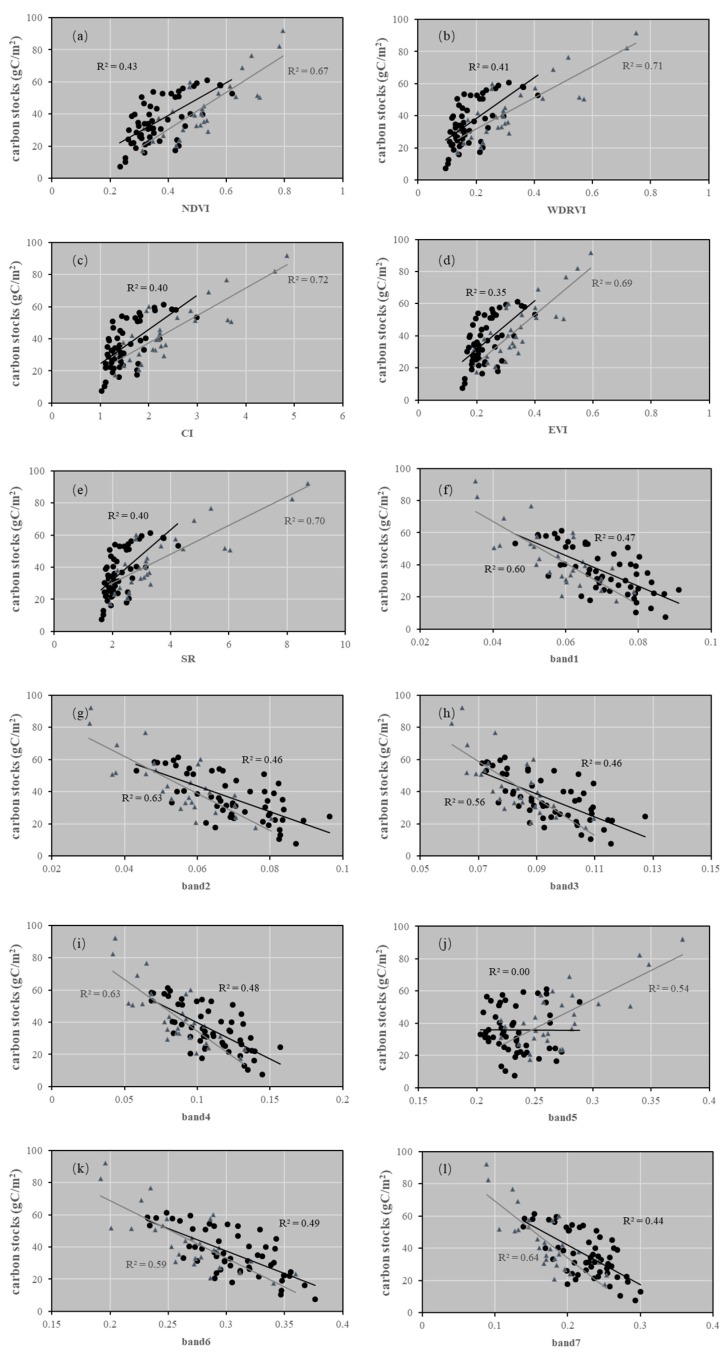
The plot-measured carbon stocks (gC/m^2^) versus 12 variables derived from the Landsat 8 OLI for the study: (**a**) carbon stocks versus the NDVI; (**b**) carbon stocks versus the WDRVI; (**c**) carbon stocks versus the CI; (**d)** carbon stocks versus the EVI; (**e**) carbon stocks versus the SR; (**f**) carbon stocks versus Band1; (**g**) carbon stocks versus Band2; (**h**) carbon stocks versus Band3; (**i**) carbon stocks versus Band4; (**j**) carbon stocks versus Band5; (**k**) carbon stocks versus Band6; (**l**) carbon stocks versus Band7. The black dots represent the typical steppe samples, and the gray triangles represent the meadow steppe samples.

Scatter plots between the image variables and carbon stocks for each grassland type are shown in Figure 2. It could be seen that Band6 had the highest correlation (R^2^ = 0.49) with the plot-measured carbon stocks of the typical steppe, followed by Band4 (R^2^ = 0.48), Band1 (R^2^ = 0.47), Band3 (R^2^ = 0.46), Band2 (R^2^ = 0.46), Band7 (R^2^ = 0.44), the NDVI (R^2^ = 0.44), the WDRVI (R^2^ = 0.41), the CI (R^2^ = 0.40), the SR (R^2^ = 0.40), the EVI (R^2^ = 0.35), and Band5 (R^2^ = 0.40). For the meadow steppe, the CI had the highest correlation (R^2^ = 0.72) with the plot-measured carbon stocks, followed by the WDRVI (R^2^ = 0.71), the SR (R^2^ = 0.70), the EVI (R^2^ = 0.69), the NDVI (R^2^ = 0.67), Band7 (R^2^ = 0.64), Band4 (R^2^ = 0.63), Band2 (R^2^ = 0.63), Band1 (R^2^ = 0.60), Band 6(0.59), Band3 (R^2^ = 0.56), and Band5 (R^2^ = 0.54). For the typical steppe, the VIs had relatively lower correlations with the plot-measured carbon stocks than did spectral bands due to the bad correlation of Band 5 (near-infrared), which is used to calculate the VIs (see Equations (1)–(5)).

### 3.3. Model Accuracy

The accuracies of the 15 models based on RK and five models based on LR were tested according to the three databases. To facilitate comparison, the highest accuracy (higher R^2^ and lower MAE and RMSE) of the three models of theoretical variograms (exponential, Gaussian, and spherical) is listed. As R^2^ cannot represent overestimation or underestimation by the model system [84], we also considered the MAE and RMSE when selecting the model with the best accuracy, the results are shown in Table 4. When the models were validated against the all-steppe samples, the model based on Band6 showed the best performance (R^2^ = 0.52, MAE = 9.07 gC/m^2^, and RMSE = 10.46 gC/m^2^) for the LR method, while for the RK method, the model based on the WDRVI showed the best performance (R^2^ = 0.68, MAE = 7.61 gC/m^2^, and RMSE = 9.67 gC/m^2^).

When the models were validated against the typical steppe samples, the model based on Band6 showed the best performance (R^2^ = 0.46, MAE = 8.47 gC/m^2^, and RMSE = 10.34 gC/m^2^) for the LR method, while for the RK method, the model based on the NDVI showed the best performance (R^2^ = 0.63, MAE = 7.04 gC/m^2^, and RMSE = 8.51 gC/m^2^), which were much higher than the corresponding values of the best LR model. When the models were validated against the meadow steppe samples, the model using the CI as the variable showed the best estimation accuracy, and the accuracy of the RK Gaussian method (R^2^ = 0.72, MAE = 8.09 gC/m^2^, and RMSE = 9.89 gC/m^2^) was higher than that of the LR method (R^2^ = 0.70, MAE = 8.99 gC/m^2^, and RMSE = 10.69 gC/m^2^).

The VI models showed more accurate results than those obtained from a single spectral band for the RK model. Upon combining the results of the most accurate models of typical steppe and meadow steppe, the RK method reaches the highest model accuracy of R^2^ = 0.69, MAE = 7.40 gC/m^2^, and RMSE = 9.01 gC/m^2^, while the LR method reaches the highest model accuracy of R^2^ = 0.53, MAE = 9.20 gC/m^2^, and RMSE = 11.10 gC/m^2^. The models based on the RK method presented an improved accuracy over that of the LR method.

### 3.4. Carbon Stock Distribution in the Study Area

**Figure 3:**
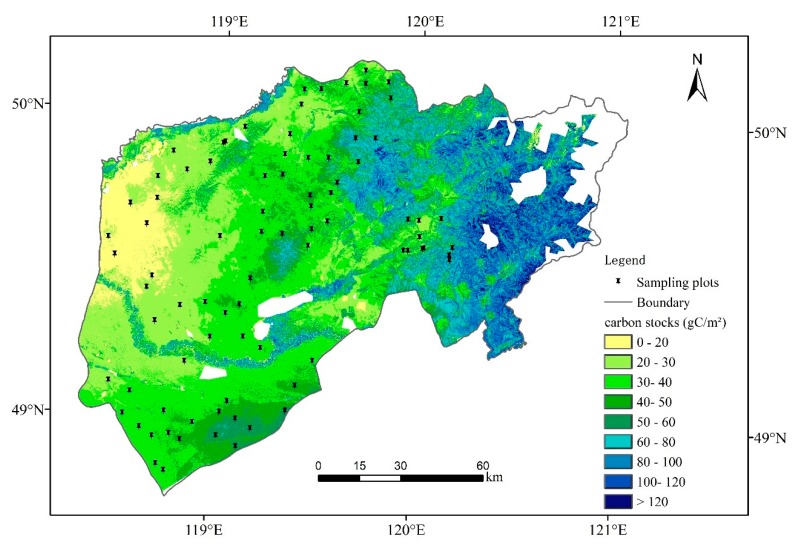
The carbon stocks spatial distribution of Chenbarhu Banner.

According to the regression and accuracy analysis, the NDVI exponential model of the RK method was used to estimate the carbon stocks of the typical steppe, while the CI spherical model of the RK method was used to estimate the carbon stocks of the meadow steppe. By mosaicking the estimated rasters of a typical steppe and meadow steppe, we finally obtained the carbon stock spatial distribution of Chenbarhu Banner (Figure 3). In general, the carbon stocks showed an increasing trend from west to east. The carbon stocks of different grassland types was shown in Table 5, the total grassland carbon stock was 79.77 × 10^4^ Mg C in the study region, and the mean density was 47.44 gC/m^2^. The maximum carbon stock density was 221.65 gC/m^2^ in the lowland steppe. The carbon stock density of the temperate meadow steppe (63.02 gC/m^2^) was much higher than those of the other steppe types. The carbon stock density of the lowland meadow was the second highest (52.75 gC/m^2^), followed by those of the temperate typical steppe (32.83 gC/m^2^) and sandy steppe (29.17 gC/m^2^). The total carbon stocks of the temperate meadow steppe account for half of the carbon stocks in the study region. The area of the temperate typical steppe is the largest (accounting for nearly 40% of the study region), and its total carbon stock was the second highest, accounting for 26.22% of that in the study region. The sandy steppe, which has the smallest area and carbon stock density, had the smallest total carbon stocks (3.39 × 10^4^ Mg C), only accounting for 4.25% of the total carbon stocks in the entire region.

### 3.5. Carbon Stock Distribution of Each Steppe Type

**Figure 4:**
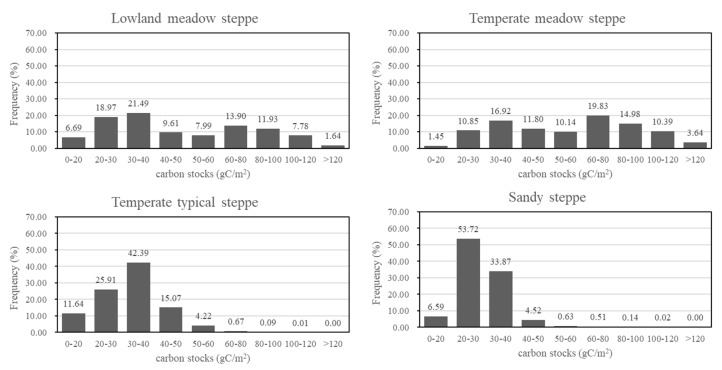
Carbon stock distribution of each steppe.

The distribution of carbon stocks was calculated for each steppe type (Figure 4). There was a similar carbon stock distribution between lowland meadow steppe and temperate meadow steppe. Over 70% of their carbon stocks were distributed from 20 to 80 gC/m^2^. There were very few areas of meadow steppe with carbon stocks of less than 20 gC/m^2^. However, 9.42% of lowland meadow steppe and 14.03% of temperate meadow steppe had carbon stocks of over 100 gC/m^2^. Most of the carbon stocks of temperate typical steppe and sandy steppe ranged from 20 to 40 gC/m^2^. Only a small amount of carbon stocks (20.6% for temperate typical steppe and 5.83% for sandy steppe) were greater than 40 gC/m^2^. Less than 1% of temperate typical steppe and sand steppe carbon stocks were higher than 60 gC/m^2^ and 50 gC/m^2^, respectively.

## 4. Discussion

### 4.1. Improvement Analysis of the RK Model

**Figure 5:**
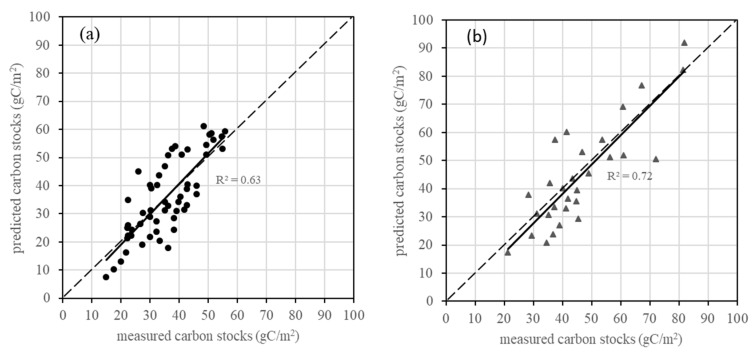

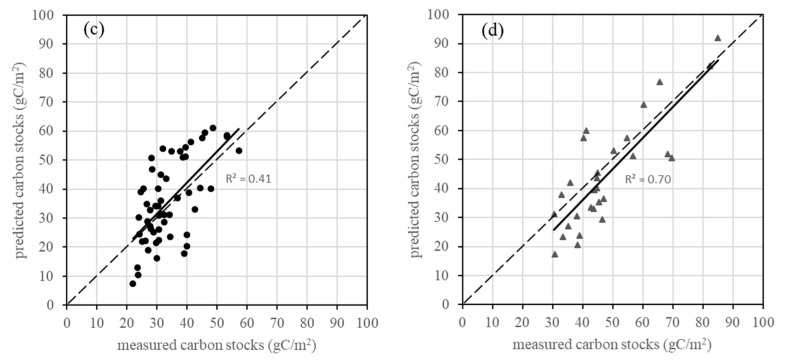
Comparison between the measured carbon stocks and the predicted grassland carbon stocks using (**a**,**b**) the RK model and (**c**,**d**) the LR model. The black dots represent the typical steppe samples, and the gray triangles represent the meadow steppe samples. The long dash lines are 1:1 line and the solid lines are linear regression line.

Figure 5 shows scatterplots of the predicted versus observed carbon stocks to show the improvement of the RK model compared with the corresponding LR model at the study site. The RK model-predicted carbon stocks were the cross-validation valuea of the NDVI exponential model (R^2^ = 0.63, MAE = 7.04 gC/m^2^, and RMSE = 8.51 gC/m^2^) and CI Gaussian model (R^2^ = 0.72, MAE = 8.09 gC/m^2^, and RMSE = 9.89 gC/m^2^) for the typical steppe and meadow steppe, respectively, and the LR model-predicted carbon stocks were the cross-validation values of the NDVI model (R^2^ = 0.41, MAE = 8.99 gC/m^2^, and RMSE = 10.92 gC/m^2^) for typical steppe and the CI model (R^2^ = 0.70, MAE = 8.99 gC/m^2^, and RMSE = 10.69 gC/m^2^) for meadow steppe. As can be seen, compared with the LR results, the accuracy of the predicted values was improved, especially for the typical steppe. The RK model improves the underestimation when carbon stocks are low (<30 gC/m^2^), but the overestimation still remains when carbon stocks are over 80 gC/m^2^.

### 4.2. Comparison between Univariate and Multivariate Regression

There are two main steppe classes in the research area: typical and meadow steppes. The two main steppe classes possess different climate conditions and vegetation statuses. Typical steppes are composed of typical drought-growing plants, which are mainly clumps of grass, accompanied by middle drought-growing hybrid grass and rhizome moss and sometimes mixed with drought-growing shrubs or small semi-shrubs. Under the conditions of moderate rain and a suitable climate, grassland vegetation is dominated by perennial caespitose grass, and root grass is called meadow steppe grass. The two steppe classes are validated separately. The best accuracy results of the models are listed in Table 4. Upon combining the results of the typical steppe (NDVI exponential model) and meadow steppe (CI Gaussian model), the univariate regression based on RK method reaches the highest model accuracy of R^2^ = 0.69, MAE = 7.40 gC/m^2^, and RMSE = 9.01 gC/m^2^. We also explored multivariate regression, the accuracy results are shown in Table 6. The best accuracy model, which used the five variables as input, had a lower accuracy (R^2^ = 0.68, MAE = 7.45 gC/m^2^, and RMSE = 9.19 gC/m^2^) than that of the combined univariate regression.

The importance of the input variables was valued according to the absolute value of the standard coefficient in the process of multiple linear regression contained in the RK model. As can be seen in Table 7, the CI contributes the most in the multivariate regression, followed by Band6, the NDVI, the WDRVI and Band7.

### 4.3. Selection of Regression Variables

In this study, vegetation indexes and spectral bands were used as regression variables, and terrain or climate factors were not selected, as the carbon stocks in the selected region were more affected by grazing behaviors, and the carbon stocks converted through the AGB were based on the residual carbon after livestock feeding. The vegetation indexes selected in this paper included the NDVI, the WDRVI, and the CI, and the and the spectral bands selected were Band6 and Band7, which were both highly correlated with the measured carbon stocks (Table 3). The NDVI is the most commonly used vegetation index, and it can reflect the background influence of the plant canopy, such as soil, wet ground, snow, dead leaves, roughness, etc., and is related to the vegetation cover. Generally, when the vegetation is dense, it approaches saturation asymptotically, and the sensitivity decreases [71,73,85,86,87,88]. This phenomenon can be seen in Figure 2, which shows that when the carbon stock reached 100 gC/m^2^, the NDVI was almost at its maximum (value of 1). However, the inversion results show that 9.42% of lowland meadow steppe and 14.03% of temperate meadow steppe have a carbon stocks over 100 gC/m^2^ (Figure 4). The WDRVI is a vegetation index established to improve the saturation of the NDVI. It is suitable for cases in which the leaf area index (LAI) is greater than 3.0 m^2^/m^2^ [70]. The CI is a vegetation index created to estimate the chlorophyll content [71,72]. According to previous research results, the LAI of a grazed meadow grassland in early July was 0.5–1.5 m^2^/m^2^, and that of a mowed meadow grassland was 2.0–3.0 m^2^/m^2^ [45,89]. The correlation analysis of carbon stocks and vegetation indexes shows that the NDVI is highly correlated with the carbon stocks of typical steppe, which may be related to the fact that the vegetation density of typical steppe is not as high. For meadow steppe, the CI showed the highest correlation with carbon stocks, followed by the WDRVI, and the relationship between the chlorophyll content and carbon stocks needs to be further studied. Band6 (1.56–1.66 microns) and Band7 (2.10–2.30 microns) are both SWIR bands and show better correlation with the carbon stocks than do other spectral bands. SWIR imaging is mainly based on the principle of target-reflected light imaging. Its imaging features are similar to those of visible light-gray images, with high contrast and clear expression of target details. There is strong light absorption by liquid water in the SWIR [90]. Early July is the period of grassland growth, when the plants are well watered, and this may be the reason for the high correlation between SWIR bands and carbon stocks.

### 4.4. Study Innovation and Limitation

In this study, Landsat8 OLI level-1 standard data products were used as data sources, and the RK method was used to predict the steppe carbon stocks in Hulunber, which produced better results compared with the LR method. There are major innovations in this paper. First, in contrast to other Landsat images, Landsat8 OLI narrows the range of the near-red light band, reduces the influence of water vapor absorption and is more conducive to the inversion of ecological parameters. Second, RK is a hybrid geostatistics method that combines the LR method with the ordinary kriging of residuals [28], thereby reducing the estimation error (see Table 4 and Figure 5). For this reason, it has also been used to map environmental variables [19,45,46,47,48,49]. In the study area, RK performs better than do the other machine learning methods (ANN and RF) in terms of predicting the grassland leaf area index across the duration of the growing season [45]. However, for many years, GIS technologies and geostatistical techniques have been developing independently, and there is a lack of user-friendly GIS environments in which to run RK, which limits the extension and application of this method [33]. There is still a lack of research on the remote-sensing inversion of vegetation carbon stocks, especially for grasslands. This study addresses the lack of research using RK to invert grassland carbon stocks. In addition, modeling typical steppe and meadow steppe separately improves the precision of the model (see Section 4.2). The study area is located in the backland of Hulunber, the northeastern part of Inner Mongolia, China. There exist few scale-up estimate of carbon stocks in the inner Monglia. Some efforts were made at larger region sacle [29,39] or another province Xilingol [91]. Our predicted model show a accuracy of R^2^ = 0.69, MAE = 7.40 gC/m^2^, and RMSE = 9.01 gC/m^2^, which is considerable with the larger region sacle [39] (R^2^ = 0.68) [29] (R^2^ = 0.66). The other research conducted in Xilingol, obtained a accuracy of R^2^ = 0.60 (meadow steppe) and 0.56 (typical steppe), were lower than our results.

This study has some limitations. First, the RK model also has great limitations, because more points are needed for kriging interpolation, and the established model cannot be used for other regions and times. This is also a common shortcoming of all empirical models. Second, there is a lack of sample points in the mountainous area of the most eastern part of the study area, where there are higher carbon stock values (Figure 3). There is a need for further improvement by incorporating newly observed data that is representative of high-carbon stock areas that were under-sampled. Third, this study is based on a field survey, which was performed on 9 July to 16 July in 2015, and it only covered one of the stages of the growth period. To obtain the dynamic changes of carbon stocks, regularly monitored ground data is needed. Therefore the next step is to explore the applicability of these methods in various stages of grassland growth. Additionally, due to the influence of soil and mixed pixels, the estimation accuracy of the carbon stocks of typical steppe is not as good as that of meadow steppe. In future research, this aspect needs to be further considered. The Sentinel-2 or Gao-fen 2 data, which were not available within the field survey time of this study and have better spatial resolutions than that of Landsat 8, can be considered. In a recent study, Sentinel-1, Landsat-8, and Sentinel-2 data were used both individually and integrally to estimate the seasonal dynamics of the LAI and AGB in a tallgrass pasture in the United States. By comparison, the integration of Sentinel-1, Landsat-8, and Sentinel-2 has the potential to improve the estimation of the LAI and AGB by more than 30% relative to the performance of the data of Landsat-8, and Sentinel-2 data with high vegetation cover (LAI > 2m^2^/m^2^, AGB > 500 g/m^2^) [92]. However, the AGB of the study area during the peak-growth stage is lower than 500 g/m^2^ [39], and the grass is not as tall as it is in the United States. Another study in irrigated grasslands showed that the use of polarimetric parameters did not improve the estimation of soil moisture and vegetation parameters (FPAR and LAI) [93], so using radar penetration to improve the estimation accuracy may not work.

## 5. Conclusions

This study explored the RK method of estimating grassland carbon stocks in northeast China. The NDVI, the WDRVI, the CI, Band6 and Band7 were used as the independent variables to build RK models separately. The results of typical steppe and meadow steppe showed the highest model accuracies of R^2^ = 0.69, MAE = 7.40 gC/m^2^, and RMSE = 9.01 gC/m^2^ via the RK method; and R^2^ = 0.53, MAE = 9.20 gC/m^2^, and RMSE = 11.10 gC/m^2^ via the LR method. The validation results show that the RK models for the five variables all have an improved accuracy over those of the LR method and the improvement in the accuracy of the model is mainly attributed to the enhancement of the estimation accuracy of the typical steppe. In general, the carbon stocks showed an increasing trend from west to east. The total grassland carbon stock in the study region was 79.77 × 10^4^ Mg C, and the mean carbon stock density was 47.44 gC/m^2^. The density decreased in the following order: temperate meadow steppe, lowland meadow steppe, temperate typical steppe, and sandy steppe. Collecting newly observed data that is representative of high-carbon stock areas and using high-spatial resolution data to explore the applicability of the RK method in various stages of grassland growth is the next step of this research.

## Figures and Tables

**Table 1:** Landsat 8 OLI data acquisition information.

Process Level	Bands Used	Path-Row	Acquisition Date
Level-1	Band1-Band7	123-25	5 July 2015
Level-1	Band1-Band7	123-26	5 July 2015
Level-1	Band1-Band7	124-25	12 July 2015
Level-1	Band1-Band7	124-26	12 July 2015

**Table 2:** Descriptive statistics of the measured carbon stocks dataset ^1^.

Grassland Types	Typical Steppe	Meadow Steppe	All Steppe
No. of samples	55	29	84
Mean	35.82	44.55	38.84
Min	7.54	17.43	7.54
Max	61.20	92.05	92.05
stdev	14.09	18.61	16.23

^1^ Carbon in units of gC/m^2^.

**Table 3:** Correlation coefficients between the image variables and carbon stocks.

	Correlation Coefficients	All Steppe (n = 84)	Typical Steppe (n = 55)	Meadow Steppe (n = 29)
Variables				
NDVI		0.731 **	0.657 **	0.820 **
WDRVI		0.736 **	0.641 **	0.840 **
CI		0.737 **	0.635 **	0.848 **
EVI		0.708 **	0.590 **	0.829 **
SR		0.723 **	0.630 **	0.836**
Band1 (coastal)		−0.731 **	−0.689 **	−0.773 **
Band2 (blue)		−0.735 **	−0.681 **	−0.792 **
Band3 (green)		−0.719 **	−0.682 **	−0.749 **
Band4 (red)		−0.746 **	−0.695 **	−0.794 **
Band5 (near-infrared)		0.471 **	−0.004	0.737 **
Band6 (SWIR 1)		−0.744 **	−0.697 **	−0.770 **
Band7 (SWIR 2)		−0.721 **	−0.662 **	−0.802 **

** refers to a significant correlation between the image variables and carbon stocks (*p* < 0.01).

**Table 4:** Validation of the RK and LR methods by leave-one-out.

Validation Samples	Variable	RK				LR		
		Model	R^2^	MAE	RMSE	R^2^	MAE	RMSE
all-steppe	NDVI	exponential	0.65	7.96	9.59	0.51	9.54	11.07
	WDRVI	exponential	0.68	7.61	9.17	0.52	9.23	11.52
	CI	exponential	0.66	7.69	9.44	0.52	9.24	11.13
	band6	exponential	0.60	8.37	10.15	0.52	9.07	10.46
	band7	spherical	0.53	9.17	11.01	0.50	9.47	10.69
typical steppe	NDVI	exponential	0.63	7.04	8.51	0.41	8.99	10.92
	WDRVI	exponential	0.64	7.10	8.63	0.39	9.30	11.31
	CI	exponential	0.60	7.39	9.17	0.38	9.38	11.36
	band6	exponential	0.57	7.68	9.20	0.46	8.47	10.34
	band7	exponential	0.45	8.59	10.50	0.42	8.84	10.74
meadow steppe	NDVI	exponential	0.63	9.72	11.37	0.63	10.57	12.04
	WDRVI	Gaussian	0.70	8.34	10.09	0.68	9.08	10.86
	CI	Gaussian	0.72	8.09	9.89	0.70	8.99	10.69
	band6	spherical	0.60	9.63	11.68	0.55	10.22	12.59
	band7	spherical	0.65	10.24	11.94	0.61	10.68	12.56

**Table 5:** The carbon stocks of different grassland types.

Grassland Types	Area (10^4^ hm^2^)	Min (gC/m^2^)	Max (gC/m^2^)	Mean (gC/m^2^)	Total (10^4^ MgC)	Proportion (%)
Lowland meadow steppe	29.36	0.00	221.65	52.75	15.49	19.42
Temperate meadow steppe	63.43	0.00	187.52	63.02	39.97	50.11
Temperate typical steppe	63.74	0.00	153.79	32.83	20.92	26.22
Sandy steppe	11.61	0.00	137.75	29.17	3.39	4.25
All-steppe	168.14	0.00	221.65	47.44	79.77	100.00

**Table 6:** The accuracy results of multivariate regression based on RK.

Variable	Model	R^2^	MRE	RMSE
NDVI, WDRVI, CI	exponential	0.68	7.70	9.24
Band6, Band7	exponential	0.61	8.32	10.14
NDVI, WDRVI, CI, Band6, Band7	exponential	0.68	7.45	9.19

**Table 7:** The importance of the variables for carbon stock prediction measured using RK.

Variable	Absolute Value of the Standard Coefficient	Ranking
CI	1.278	1
Band6	0.572	2
NDVI	0.553	3
WDRVI	0.495	4
Band7	0.003	5
